# A Noise Reduction Method for Signal Reconstruction and Error Compensation of a Maglev Gyroscope Under Persistent External Interference

**DOI:** 10.3390/s24248005

**Published:** 2024-12-15

**Authors:** Di Liu, Zhen Shi, Ziyi Yang, Chenxi Zou

**Affiliations:** School of Geology Engineering and Geomatics, Chang’an University, 126 Yanta Road, Xi’an 710054, China; di.liu@chd.edu.cn (D.L.); 2022226021@chd.edu.cn (Z.Y.); 2023126023@chd.edu.cn (C.Z.)

**Keywords:** maglev gyroscope, azimuth measurement, adaptive particle swarm optimization algorithm, variational modal decomposition, trend term extraction, noise reduction

## Abstract

To eliminate the noise interference caused by continuous external environmental disturbances on the rotor signals of a maglev gyroscope, this study proposes a noise reduction method that integrates an adaptive particle swarm optimization variational modal decomposition algorithm with a strategy for error compensation of the trend term in reconstructed signals, significantly improving the azimuth measurement accuracy of the gyroscope torque sensor. The optimal parameters for the variational modal decomposition algorithm were determined using the adaptive particle swarm optimization algorithm, allowing for the accurate decomposition of noisy rotor signals. Additionally, using multi-scale permutation entropy as a criterion for discriminant, the signal components were filtered and summed to obtain the denoised reconstructed signal. Furthermore, an empirical mode decomposition algorithm was employed to extract the trend term of the reconstructed signal, which was then used to compensate for the errors in the reconstructed signal, achieving significant noise reduction. On-site experiments were conducted on the high-precision GNSS baseline of the Xianyang Yuan Tunnel in the second phase of the project to divert water from the Han River to the Wei River, where this method was applied to process and analyze multiple sets of rotor signals. The experimental results show that this method effectively suppresses continuous external environmental interference, reducing the average standard deviation of the compensated signals by 46.10% and the average measurement error of the north azimuth by 45.63%. Its noise reduction performance surpasses that of the other four algorithms.

## 1. Introduction

High-precision azimuthal references play a crucial role in ensuring the safety, efficiency, economy, and environmental friendliness of underground engineering [1,2]. As an inertial measurement device, the gyroscope theodolite can provide accurate azimuth measurement data in underground or indoor environments, where external signals like GNSS are unavailable. For this reason, it has been widely used in various industries, such as tunnel construction, mining operations, weapon navigation, and geological surveys [3].

Traditional gyroscope theodolites measure the azimuth by dynamically tracking the meridian to sense the pointing torque caused by the Earth’s rotation [4]. However, during this oscillating measurement process, the data of the pointing torque are challenging to preserve, and subsequent data processing cannot be carried out, which severely restricts the reliability of the azimuth measurement results. Unlike the north-seeking principle of traditional instruments, the magnetic suspension gyroscope theodolite uses a static torque balance principle for azimuth measurement [5]. The specific operation is to apply a reverse torque to the sensitive part of the gyroscope to achieve a balanced state with the pointing torque and to measure the magnitude of this reverse torque for precise azimuth measurement. Compared to traditional instruments, the magnetic suspension gyroscope theodolite can more objectively and accurately capture the subtle changes in the pointing torque during orientation. After the magnetic levitation gyroscope has completed a full cycle of data sampling, we can enhance the precision and reliability of the gyroscope’s orientation through detailed analysis and processing of the torque sensor signals. This provides a more accurate azimuthal reference for underground engineering construction.

The torque sensor is the core component of the magnetic suspension gyroscope theodolite for azimuth measurement. This sensor accurately collects stator and rotor signals to obtain reverse torque data and then calculates the azimuth result [6]. However, during the signal collection process of the torque sensor, interference from external environmental factors often leads to noise in the sampled signals, causing deviations between the instrument’s azimuth measurement results and the actual azimuth [7]. Advanced filtering techniques are typically used to denoise the sampled signals from the torque sensor to improve the directional accuracy of the instrument, ensuring the precision and reliability of the azimuth measurement results.

Previous research has shown that changes in the external environment have a minimal impact on the stability of the sensor’s stator signals. In contrast, the interference with the rotor signals is relatively significant [8]. Li Huiru used the Vondrak method to smooth the rotor signals, reducing glitches in the signal sequence and significantly decreasing the root mean square error of the reconstructed signal [9]. Qin Peng proposed an adaptive fractal filtering algorithm based on the principle of fractional difference estimation, effectively reducing the dispersion of the rotor signals [10]. However, due to the non-stationary, nonlinear, and time-varying characteristics of the rotor signals, noise reduction methods such as Vondrak and fractal filtering are challenging in effectively improving the instrument’s azimuth measurement’s precision. Ma Ji employed an optimized wavelet transform (OWT) method to process the rotor signals, using wavelet functions of different scales to treat the signals. The results demonstrated the effectiveness of the OWT algorithm in signal noise reduction [11]. However, the noise reduction effect of the OWT method is greatly influenced by parameter settings and lacks adaptability, leading to uncertainty in the algorithm’s noise reduction performance. Zhang Xuewei optimized the Hilbert–Huang transform (OHHT) method, adaptively decomposing the signal into multiple intrinsic mode functions based on the signal’s time-scale characteristics. Noise reduction of the rotor signals was achieved by reconstructing the intrinsic modal function (IMF) components [12]. However, OHHT has drawbacks such as endpoint effects and mode mixing, making it challenging to ensure the accuracy of rotor signal decomposition under external environmental interference. Wang Yiwen used the heuristic segmentation algorithm (HSA) and the two-sample Kolmogorov–Smirnov test method to denoise rotor signals affected by instantaneous interference torque, effectively eliminating jump sequences in the signals [13]. However, due to the HSA method’s inability to identify the baseline sequence of the rotor signals under the influence of continuous interference torque, it has certain limitations in practical applications. Among various noise reduction algorithms, OWT and OHHT stand out with their robust noise reduction capabilities and have become the most widely adopted techniques for processing rotor signals in complex environments.

In 2014, Dragomiretskiy and others proposed the variational mode decomposition (VMD) algorithm, a signal decomposition algorithm based on variational problems which determines modal functions through an iterative optimization process [14]. Compared with traditional signal decomposition algorithms, the VMD algorithm has stronger robustness and rigorous theoretical support. In recent years, the VMD algorithm has been widely applied in many fields, including mechanical fault diagnosis, biomedical signal processing, communication signal analysis, and more [15,16]. In the VMD algorithm, the number of modes and the penalty factor are two crucial parameters that significantly affect the algorithm’s performance [17]. To enhance the reliability of signal decomposition in specific application scenarios using the VMD algorithm, researchers have applied heuristic optimization algorithms such as particle swarm optimization (PSO), the genetic optimization algorithm (GA), and the whale optimization algorithm (WOA) to the optimization of VMD parameters [18,19,20]. Among these optimization algorithms, GA is a heuristic search strategy that draws on the concepts of natural selection and genetics, aiming to address various optimization and search challenges. The WOA draws wisdom from the social interactions and foraging strategies of humpback whales in the natural world, presenting an innovative and efficient optimization method. The PSO mimics the group behavior of birds and fish, relying on the cooperation and information exchange among individuals to collectively seek the best solution to a problem. In particular, the PSO algorithm stands out due to its excellent global search capability, fewer adjustment parameters, and higher computational efficiency, especially when dealing with parameter optimization issues in large data volumes, where it has more advantages than other optimization algorithms.

In response to the issue of mixed noise in the rotor signal under continuous external environmental interference, which affects the accuracy of the maglev gyroscope torque sensor’s orientation measurement, this study proposes a noise reduction method that integrates an adaptive particle swarm optimization variational mode decomposition (APVMD) algorithm with a trend term error compensation strategy for signal reconstruction. The method first adopts dispersion entropy as the fitness function, utilizing the adaptive particle swarm optimization (APSO) algorithm to precisely determine the optimal parameters for VMD. Subsequently, a criterion for modal component selection based on multi-scale permutation entropy is established, performing preliminary noise reduction on the modal components obtained from the parameter-optimized VMD to obtain the reconstructed signal. Finally, to further reduce the overall offset of the rotor signal caused by continuous external environmental interference, this study extracts the trend term from the reconstructed signal and compensates for the error of the reconstructed signal based on the mean of the trend term, thus obtaining the final compensated signal. Through comparative analysis with other algorithms, the noise reduction method proposed in this study demonstrates its effectiveness and superiority in processing rotor signals subject to continuous external environmental interference. This research provides a new perspective for the field of rotor signal processing. It significantly improves the orientation measurement accuracy of the maglev gyroscope, promoting the advancement of engineering measurement technology in confined spaces.

## 2. Principles of Azimuth Measurement and Error Analysis for Maglev Gyroscope Torque Sensors

### 2.1. Azimuth Measurement Principle

The high-speed rotating gyroscope generates a pointing torque M under the action of the Earth’s rotation. The magnitude and direction of this torque continuously change as the Earth’s rotation persists [21].

(1) $$M=H\times \omega_{E}\cos\varphi \sin\alpha_{T}$$

where H represents the angular momentum of the gyroscope’s rotation, $\omega_{E}$ is the angular velocity of the Earth’s rotation, φ is the local latitude, and $\alpha_{T}$ is the azimuth angle of the gyroscope relative to North.

To calculate the magnitude of the pointing torque, the torque feedback control system applies an opposing torque of equal magnitude but opposite direction to the sensitive part of the gyroscope in the horizontal direction [22]. By adjusting the intensity of the input current, the system continuously adjusts until the sensitive part of the gyroscope reaches a torque balance state. At the moment the gyroscope achieves torque balance, the torque sensor begins to record a series of discrete dynamic photoelectric parameters, including rotor signal and stator signal.

The system is preset to collect N = 20,000 sets of rotor and stator signal data at each balance position [23]. Since these signal data are obtained from independent observations, the average torque $M_{mean}^{\prime }$ at the current balance position can represent the opposing torque at that position.

(2) $$M_{mean}^{\prime }=\frac{k\sum_{i=1}^{N}I_{Ri}I_{Si}}{N}$$

where k is the torque actuator coefficient, $I_{Ri}$ is the rotor signal value, and $I_{Si}$ is the stator signal value.

Subsequently, based on the torque balance equation $M_{mean}^{\prime }=M$, the gyroscope torque sensor’s orientation measurement result can be obtained, which is the gyroscope’s north azimuth angle.

(3) $$\alpha_{T}=\arcsin\frac{k\sum_{i=1}^{N}I_{Ri}I_{Si}}{NH\omega_{E}\cos\varphi }$$

### 2.2. Analysis of Rotor Signal Error Under Continuous Disturbance of External Environment

**Figure 1:**
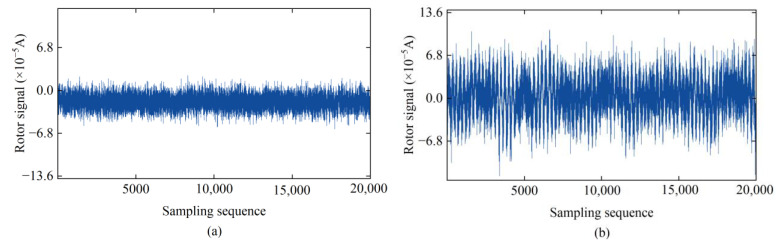
Time series of rotor sampling signals in different observation environments: (**a**) stable observation environment; (**b**) observation environment with persistent external interference.

In an ideal observation environment, the time series of the rotor signal in the gyroscope torque sensor exhibits a stationary state (Figure 1a), which allows for precise orientation. However, during the actual observation process, it is inevitable to encounter multiple environmental interferences, such as wind-induced vibration disturbances and construction-related vibrations. These factors result in noise mixing into the signals collected by the torque sensor. Since the rotor system undergoes suspension and drops along with the sensitive part of the gyroscope during the signal collection process, the rotor signal is highly sensitive to environmental changes. Under the continuous influence of the external environment, the equilibrium position of the gyroscope rotation axis is constantly disturbed by torque throughout the entire signal sampling interval, causing the rotor signal to exhibit significant non-linear and non-stationary characteristics. In local regions, the signal exhibits pronounced white noise characteristics, whereas overall, it is accompanied by an apparent trend-like low-frequency system noise (Figure 1b).

**Figure 2:**
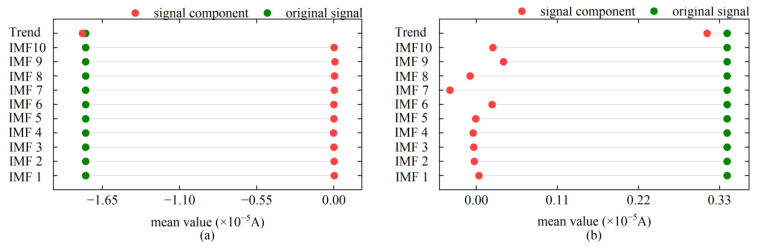
Mean distribution of rotor signal and its decomposed components under different observation environments: (**a**) stable observation environment; (**b**) observation environment with persistent external interference.

The sensitivity of the rotor signal to the environment and its response characteristics to disturbances often cause its expected value to deviate from the actual north direction, leading to significant deviations in the azimuth measurement results [24]. Therefore, accurately obtaining the expected value of the rotor signal is the core of compensating for the overall offset error of the signal. By observing the mean distribution characteristics of the rotor signal’s empirical mode decomposition (EMD) results shown in Figure 2, we can find that, compared to the IMF components, the mean of the trend term is closer to the expected value of the rotor signal. This high consistency between the trend term mean and the rotor signal expected value is not affected by the signal type. Further practical calculations indicate that for rotor signals that have undergone sufficient noise reduction processing, the difference between the azimuth calculation results using the trend term mean as the expected value and the original output results of the gyroscope torque sensor is almost negligible. Therefore, by effectively reducing the noise of the rotor signal and extracting its trend term, we essentially obtain the accurate expected value of the rotor signal. On this basis, by compensating for the overall offset error of the rotor signal, the disturbed data can be restored to an ideal state, thereby ensuring that the maglev gyroscope torque sensor can maintain high-precision orientation capabilities under continuous external environmental interference.

## 3. Method

### 3.1. Basic Principles of the Variational Mode Decomposition Algorithm

VMD is a signal decomposition algorithm that can decompose an input signal f(t) into several IMF components [25]. These IMF components can be described as amplitude-modulated frequency-modulated signals, with the following expression:

(4) $$\mu_{k}(t)=A_{k}(t)\cos(\Phi_{k}(t))$$

where $A_{k}(t)$ represents the amplitude envelope of $\mu_{k}(t)$, and $\Phi_{k}(t)$ represents the instantaneous phase of $\mu_{k}(t)$.

The corresponding one-sided spectrum is obtained by performing the Hilbert transform on the modal functions, which can further yield the baseband signal. Next, the $L^{2}$ norm of the gradient of this baseband signal is calculated, thereby obtaining the bandwidth of each component. The corresponding constrained variational model is:

(5) $$\left\{\begin{array}{l} \underset{\left\{h_{k}\right\},\left\{\omega_{k}\right\}}{\min}\left\{{\sum_{k=1}^{K}\left\|\partial_{t}{\left[\left(\delta (t)+\frac{j}{\pi t})\mu_{k}(t\right)\right]}^{e^{-j\omega_{k}t}}\right\|}_{2}^{2}\right\}\\ \mathrm{subject}\;\mathrm{to}\;\sum_{k=1}^{K}\mu_{k}(t)=f(t) \end{array}\right.$$

where K represents the number of modal functions, $\mu_{k}$ is the modal function, and $\omega_{k}$ is the central frequency of the frequency component corresponding to each modal function.

To find the optimal solution for the constrained variational problem, it is converted into an unconstrained variational problem. Therefore, a Lagrange function and a quadratic penalty factor are introduced, and the expression for this function is:

(6) $$L(\left\{\mu_{k}\right\},\left\{\omega_{k}\right\},\lambda )=\alpha \sum_{k=1}^{K}{\left\|\partial_{t}{\left[\left(\delta (t)+\frac{j}{\pi t})\mu_{k}(t\right)\right]}^{e^{-j}\omega_{k}t}\right\|}_{2}^{2}+{\left\|f(t)-\sum_{k=1}^{K}\mu_{k}(t)\right\|}_{2}^{2}+\langle \lambda (t),f(t)-\sum_{k=1}^{K}\mu_{k}(t)\rangle$$

where α is the quadratic penalty factor, and λ(t) is the Lagrange multiplier.

Using the alternating direction method of multipliers to solve the constrained variational problem, the saddle point of the Lagrangian expression is found by alternately updating $\mu_{k}^{n+1}$, $\omega_{k}^{n+1}$, and $\lambda^{n+1}$.

(7) $${\hat{\mu }}_{k}^{n+1}(\omega )=\frac{\hat{f}(\omega )-\sum_{i\neq k}{\hat{\mu }}_{k}^{n}(\omega )+\frac{{\hat{\lambda }}^{n}(\omega )}{2}}{1+2\alpha {\left(\omega -\omega_{k}^{n}\right)}^{2}}$$

(8) $$\omega_{k}^{n+1}=\frac{\int_{0}^{\infty }\omega {\left|{\hat{\mu }}_{n}^{k}(\omega )\right|}^{2}d\omega }{\int_{0}^{\infty }{\left|{\hat{\mu }}_{n}^{k}(\omega )\right|}^{2}d\omega }$$

(9) $$\lambda^{n+1}(\omega )=\lambda^{n}(\omega )+\tau (f(\omega )-\sum_{k=1}^{K}\mu_{k}^{n+1}(\omega ))$$

where ${\hat{\mu }}_{k}^{n+1}(\omega )$, $\hat{f}(\omega )$, ${\hat{\mu }}_{i}^{n}(\omega )$, ${\hat{\lambda }}^{n}(\omega )$ are the Fourier transforms of $\mu_{k}^{n+1}(t)$, f(t), $\mu_{i}^{n}(t)$, $\lambda^{n}(t)$, respectively; n is the number of iterations, and τ is the time step of λ.

Continuously update $\mu_{k}^{n+1}$, $\omega_{k}^{n+1}$, $\lambda^{n+1}$ to satisfy the constraint conditions:

(10) $$\sum_{k=1}^{K}\frac{{\left\|\mu_{k}^{n+1}(\omega )-\mu_{k}^{n}(\omega )\right\|}_{2}^{2}}{{\left\|\mu_{k}^{n+1}(\omega )\right\|}_{2}^{2}}<\varepsilon$$

where ε is the convergence parameter, which is generally taken as 1 × 10^−7^.

According to the principle of the VMD algorithm mentioned above, parameters K and α, as two key hyperparameters, have a significant impact on the decomposition results [26]. If the value of K is too small, it may lead to the IMF components having too wide a bandwidth, unable to effectively separate different frequency components in the signal; conversely, if the value of K is too large, it may result in the signal being over-smoothed, losing crucial detailed information. Similarly, if the value of α is set too small, it will not be able to accurately separate frequency components; while if the value of α is too large, it may lead to frequent updates of the center frequencies, affecting the stability of the IMF components. Since there are no universal criteria to determine the optimal values of K and α, the selection of these two parameters usually depends on the specific experimental conditions and the operator’s experience.

### 3.2. Particle Swarm Optimization Algorithm

The PSO algorithm is an optimization technique based on the principle of swarm intelligence, first proposed by Kennedy and Eberhart in 1995 [27]. The algorithm draws on the social behavior patterns of bird flocks, searching for the optimal solution to a problem through collaboration and information exchange among individuals within the group. PSO algorithm is widely used in various optimization fields due to its simplicity, ease of implementation, and excellent global search capability [28,29,30].

In the initial phase of the PSO algorithm, the algorithm randomly initializes the positions and velocities of a group of particles in the solution space. These particles represent potential solutions to a given problem. The algorithm uses the fitness function value as the evaluation criterion, with each particle associated with a fitness function value that reflects the quality of the solution represented by the particle. To guide the entire particle swarm toward the optimal position of the fitness function, particles interact with each other through information exchange, influencing each other.

For each particle in the swarm, the algorithm compares its current position with its historical best position (*pbest*). If the current position is superior, then the particle’s *pbest* is updated. Subsequently, the algorithm compares the *pbest* of all particles with the current global best position (*gbest*), and updates *gbest* based on the comparison results.

The update formulas for particle velocity $v_{i}$ and position $x_{i}$ are as follows:

(11) $$v_{i}(t+1)=\omega v_{i}(t)+c_{1}r_{1}(pbest_{i}-x_{i}(t))+c_{2}r_{2}(gbest-x_{i}(t))$$

(12) $$x_{i}(t+1)=x_{i}(t)+v_{i}(t+1)$$

where ω is the inertia weight, $c_{1}$ and $c_{2}$ are the individual learning factor and the social learning factor, respectively, and $r_{1}$, $r_{2}$ are random numbers uniformly distributed in the interval [0, 1].

During the iteration process, each particle continuously updates its velocity and position, and this process continues until the predetermined number of iterations is reached or the improvement of the global optimal solution falls below a specific threshold. When the algorithm terminates, the fitness function value corresponding to the global optimal position is the optimal value, and the particle position corresponding to this optimal value is the optimal solution to the problem.

Although the PSO algorithm is simple and efficient, it may face challenges such as local optima and parameter sensitivity when dealing with certain complex problems [31]. To overcome these drawbacks, this paper performs a linear adjustment of the inertia weight and learning factors that vary with the number of iterations. Specifically, by adjusting the inertia weight, it helps to shift the focus of the search from global to local search at the later stages of the algorithm’s iteration; and by adjusting the learning factors, the step size of particles moving towards their individual best position and the global best position can be controlled, respectively. The adaptive search process of the PSO algorithm is implemented through the following formulas.

(13) $$\left\{\begin{array}{l} \omega =\omega_{\max}-(\omega_{\max}-\omega_{\min})\frac{iter}{T}\\ c_{1}=c_{\max}-(c_{\max}-c_{\min})\frac{iter}{T}\\ c_{2}=c_{\min}-(c_{\max}-c_{\min})\frac{iter}{T} \end{array}\right.$$

where $\omega_{\min}$ and $\omega_{\max}$ are set to 0.5 and 1.4, respectively, $c_{\min}$ and $c_{\max}$ are set to 1 and 2, respectively, iter is the current iteration number, and T is the maximum number of iterations.

In practical applications, the maximum number of iterations is often related to the complexity of the target signal, the performance of the algorithm, and the choice of the fitness function. Generally, the maximum number of iterations can be set to 20 or determined through multiple simulation experiments to find an appropriate maximum number of iterations. In this research, the results of simulation experiments on the maximum number of iterations show that during the parameter optimization process for rotor signal decomposition, the fitness function values corresponding to the APSO, GA, and WOA algorithms usually stabilize after about 10 iterations (not more than 13 at most), and no significant fluctuations are observed thereafter. Therefore, setting the maximum number of iterations to 15 is a reasonable and prudent choice.

Given the characteristics of the rotor signal, we have carefully configured other relevant parameters of the PSO algorithm. The specific settings are as follows: the size of the particle swarm is set to 500, the search range for the number of modal functions K is from 2 to 15, and the search range for the quadratic penalty factor α is from 100 to 8000. Given the difference in the search range for the number of modal functions and the quadratic penalty factor, we set the maximum velocity of the particles to 2 and 500, respectively. In addition, dispersion entropy, as a technique for analyzing the complexity and randomness of time series, has been widely used in various fields such as biomedical signal processing, financial time series analysis, and mechanical fault diagnosis due to its excellent nonlinear analysis capabilities and anti-interference characteristics. Dispersion entropy can effectively quantify the complexity and randomness of time series, thus comprehensively analyzing the nonlinear and non-stationary characteristics of rotor signals. Therefore, this paper uses it as the fitness function and adopts the APSO algorithm to optimize the values of parameters K and α.

### 3.3. Signal Trend Term Extraction Method

The EMD algorithm can transform non-stationary time series into a series of mutually independent sub-sequences with varying characteristic scales [32,33]. These sub-sequences are called IMF components. The IMF components must satisfy two conditions: (1) the number of extreme points should be equal to the number of zero-crossings, or at most differ by one; (2) the average of the upper envelope formed by the maximum points and the lower envelope formed by the minimum points should both be equal to zero.

The steps for decomposing the original time series s(t) using the EMD algorithm are as follows:

Step 1: Identify all the extreme points of s(t), and fit the maximum value envelope $e_{+}(t)$ and the minimum value envelope $e_{-}(t)$ of the signal using cubic spline functions.

Step 2: Calculate the mean m(t) of the upper and lower envelopes, and compute the difference h(t) between s(t) and m(t).

(14) $$m(t)=\frac{e_{+}(t)+e_{-}(t)}{2}$$

(15) h(t)=s(t)−m(t)

Step 3: If h(t) does not satisfy the two constraint conditions of IMF, repeat steps 1–2 for h(t) until the conditions are met; if h(t) satisfies the two constraint conditions of IMF, then h(t) is taken as the first IMF1 of s(t). The difference between s(t) and IMF1 is $R_{1}(t)$:

(16) $$R_{1}(t)=s(t)-IMF_{1}$$

Step 4: Take $R_{1}(t)$ as the new original time series and repeat steps 1–3 to obtain the remaining IMF components and the final residual component $R_{n}(t)$.

(17) $$s(t)=\sum_{i=1}^{n}IMF_{i}+R_{n}(t)$$

where n represents the number of IMF components and $R_{n}(t)$ is the trend term, which represents the average trend or mean of the signal.

The EMD algorithm decomposes the data based on their own characteristics without the need for any presetting of basic functions or parameters. This method can effectively reveal the features of the signal at different time scales, thus demonstrating outstanding performance in signal trend extraction [34,35,36].

### 3.4. The Specific Steps of the Rotor Signal Denoising Method

**Figure 3:**
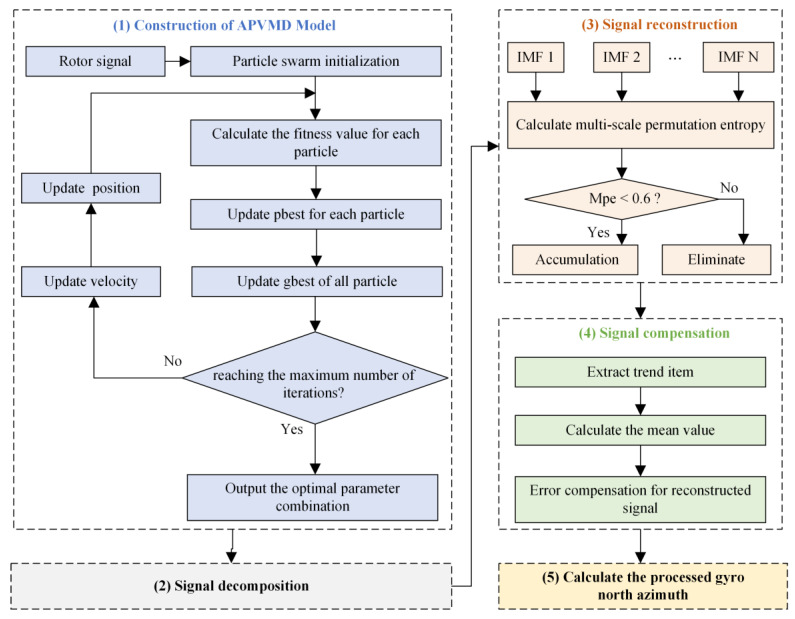
The framework of rotor signal denoising algorithm integrating APVMD signal decomposition and reconstructed signal error compensation.

This study proposes a noise reduction method that combines the APVMD algorithm with reconstructed signal error compensation. This method consists of the following five steps, and its basic process is shown in Figure 3.

(1) Constructing the APVMD model: Firstly, initialize the state of the particle swarm, where the position of each particle represents a configuration of a set of K and α parameters. Perform VMD on the rotor signal, and determine the optimal fitness function of the particle swarm by comparing the dispersion entropy corresponding to each particle. Continuously update the particle velocity, position, and iterate the best fitness function until the preset number of iterations is reached. After the iteration, the particle position corresponding to the optimal fitness function is the optimal parameter combination.
(2) Rotor signal decomposition: The APVMD is used to decompose the rotor signal into IMF components with different center frequencies.
(3) Signal component reconstruction: Using multi-scale sample entropy as the discriminant criterion, the noise IMF components are removed, and the remaining signal IMF components are accumulated to reconstruct the signal, denoted as APVMD-r. At this point, most of the high-frequency noise in the reconstructed signal has been effectively removed.
(4) Reconstructed signal error compensation: Perform EMD on APVMD-r, and the extracted residual component accurately reflects the accurate trend information of the reconstructed signal. Calculate the mean of the trend term of the reconstructed signal and compensate for the error of the reconstructed signal to correct its overall offset, thereby obtaining the final compensated signal APVMD-c. At this point, the noise reduction processing of the rotor signal is completed.
(5) Calculation of the gyroscope’s north azimuth: According to the principle of orientation measurement by the gyroscope torque sensor, the compensated signal APVMD-c is considered as the filtered rotor signal and substituted into Formula (3) for calculation, thereby obtaining the processed gyroscope’s north azimuth.

## 4. Results and Analysis

### 4.1. Experimental Data and Evaluation Indicators

**Figure 4:**
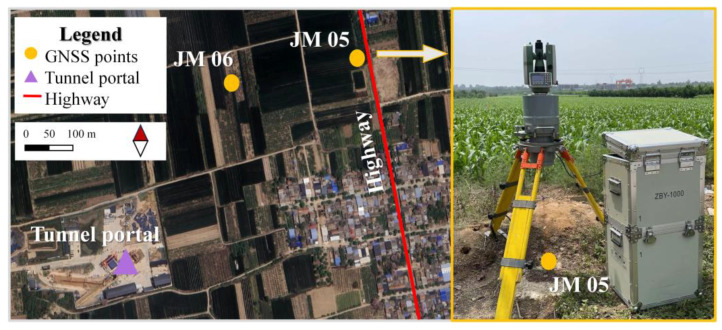
Schematic diagram of the field experiment.

To verify the effectiveness of our method, we conducted a north azimuth measurement experiment at the ground control point JM05 of the Xianyangyuan Tunnel Shield Section in the second phase of the Hanjiang to Weihe Water Diversion Project. The Xianyangyuan Tunnel is 34 km long and is an essential thoroughfare of the northern main line. As shown in Figure 4, a gyroscope was set up at point JM05 to measure the north azimuth of the GNSS baseline JM05-JM06. During the measurement, the instrument was exposed to environmental wind vibration disturbances and vibration interference caused by road traffic, resulting in the rotor current signals mixed with continuous noise interference. On the baseline JM05-JM06, we collected 15 groups of rotor signals continuously disturbed by the external environment, with each group of signals sampled for 8 min. Each group of rotor signals consisted of 20,000 discrete rotor current values collected by the gyroscope torque sensor system.

The maglev gyroscope theodolite was set up within a high-precision GNSS control network in this experiment. Since the north azimuth accuracy measured by the GNSS control network was better than 5″, which far exceeded the azimuth measurement accuracy of the instrument itself, we could use the difference *D* between the north azimuth of the rotor signal before and after noise reduction and the north azimuth measured by GNSS as the standard for evaluating external conformity accuracy. The smaller the *D* value, the higher the precision of the gyroscope azimuth measurement results. At the same time, we used the standard deviation (*Std*) as an indicator to evaluate the internal conformity accuracy of the denoised signal, in order to compare the discrete degree of the signal before and after noise reduction. The smaller the *Std* value, the better the stability of the rotor signal. The following are the specific calculation formulas for the evaluation of indicators *D* and *Std*:

(18) $$D=\left|\arctan\frac{k\sum_{i=1}^{N}I_{Si}I_{Ri}^{\prime }}{NH\omega_{e}\cos\varphi }-\alpha_{0}\right|$$

where $I_{Ri}^{\prime }$ represents the rotor signal after noise reduction, and $\alpha_{0}$ represents the GNSS north azimuth.

(19) $$Std=\sqrt{\frac{\sum_{i=1}^{N}{\left(I_{Ri}-{\bar{I}}_{Ri}\right)}^{2}}{N-1}}$$

### 4.2. Rotor Signal Noise Reduction Processing and Result Analysis

**Figure 5:**
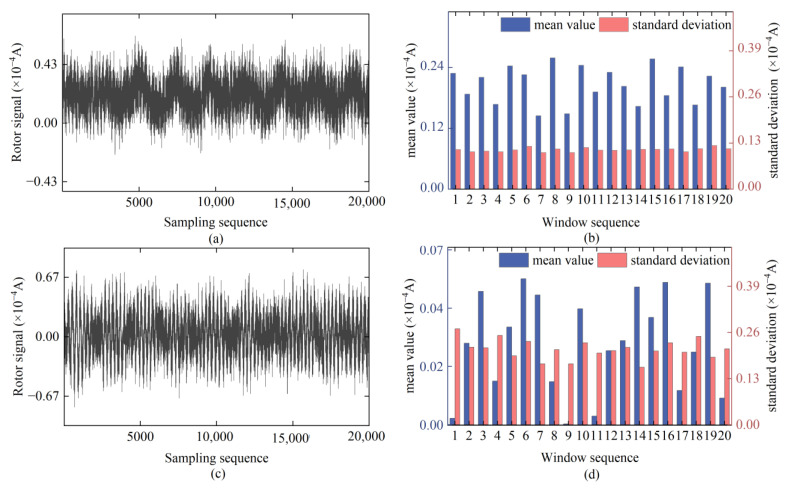

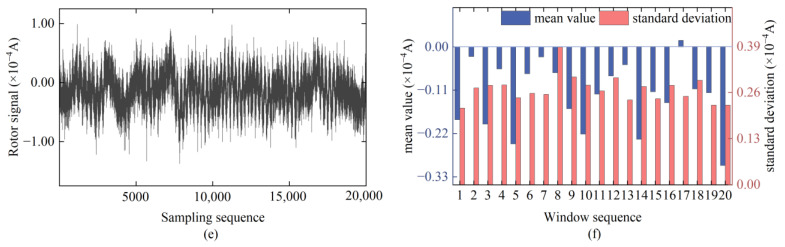
Typical classification and data characteristics of rotor signals under persistent external environmental interference: (**a**,**b**) irregular periodic-type; (**c**,**d**) jitter-type; (**e**,**f**) mixed-type.

Under continuous interference from the external environment, we conducted an in-depth analysis of the time series data from the 15 sets of rotor signals that were collected. Based on their waveform characteristics, these signals can be categorized into three types: irregular periodic (Figure 5a), jitter (Figure 5c), and mixed (Figure 5e).

As shown in Figure 5b, the Std of the irregular periodic-type rotor signals remains generally stable throughout the sampling process, but their mean value exhibits irregular fluctuations. This instability causes the rotor signals to deviate from the actual values. As shown in Figure 5d, both the mean value and Std of the jitter-type rotor signals show unstable trends throughout the sampling sequence. Compared to the irregular periodic type, the jitter-type rotor signals have a more severe degree of dispersion and a more significant fluctuation range of the mean value, which alters the overall trend of the rotor signals. As shown in Figure 5f, the mixed-type rotor signals possess both irregular periodicity and jitter characteristics. The jitter feature is quite pronounced within the sampling interval from 7000 epochs to 14,000 epochs, with both the mean value and Std showing significant jumps. These analysis results indicate that interference from the external environment leads to a decrease in the mean value stability and an increase in the degree of dispersion of the rotor signals, resulting in errors in the sampled signals and further affecting the reliability of the orientation results.

Owing to space constraints, we will select one set of signals from each type of rotor signal noise reduction process for detailed explanation. In the subsequent content, we will refer to these three sets of example signals as Signal 1, Signal 2, and Signal 3, respectively.

**Figure 6:**
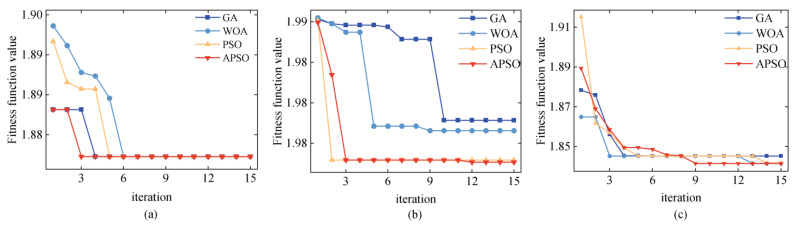
Iteration process of fitness function values corresponding to different optimization algorithms: (**a**) Signal 1; (**b**) Signal 2; (**c**) Signal 3.

In this study, to verify the effectiveness of the APSO algorithm, we employed the GA, WOA, PSO, and APSO algorithms to determine the parameters of VMD. As shown in the fitness function iteration process in Figure 6, the APSO algorithm rapidly converges to stability after the 3rd, 12th, and 9th iterations, accurately locating the optimal fitness function value. Compared with the other three parameter optimization algorithms, the APSO algorithm demonstrates the best performance in terms of convergence speed, optimization precision, and the ability to escape from local optima.

**Figure 7:**
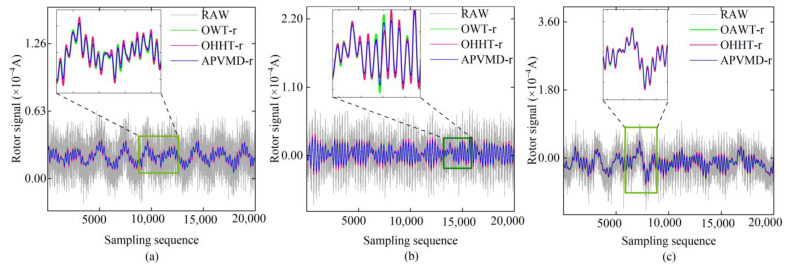
Reconstructed results of rotor signals: (**a**) reconstructed signal corresponding to Signal 1; (**b**) reconstructed signal corresponding to Signal 2; (**c**) reconstructed signal corresponding to Signal 3.

Through the particle position corresponding to the minimum fitness function value, we determined the optimal parameter combination for the decomposition of rotor signals using the APVMD algorithm: for Signal 1, the optimal parameters are K = 9, α = 5891; for Signal 2, the optimal parameters are K = 10, α = 6703; and for Signal 3, the optimal parameters are K = 10, α = 7000. Accordingly, after APVMD processing, Signal 1 was adaptively decomposed into 9 IMF components, while Signal 2 and Signal 3 were decomposed into 10 IMF components. Furthermore, based on the criterion for identifying the dominant signal components (that is, IMF components with a multi-scale permutation entropy less than 0.6 are considered stable), we identified them as the practical IMF components of the signal, which contain the measurement information of the reverse torque. According to the results of multi-scale permutation entropy calculations shown in Table 1, we selected the components IMF9, IMF9-IMF10, and IMF9-IMF10 for superposition, obtaining the three sets of reconstructed signals shown in Figure 7 (marked as “method-r”). The results in Figure 7 indicate that compared to the original untreated rotor signals, the OWT, OHHT, and APVMD algorithms significantly reduced the degree of dispersion of the signals, and most of the high-frequency noise in the rotor signals was effectively filtered out. From the zoomed-in sections of Figure 7, it can be seen that APVMD-r performed the best in terms of improving signal mutations and noise suppression, with the smoothness of its reconstructed signal superior to that of OWT-r and OHHT-r.

**Figure 8:**
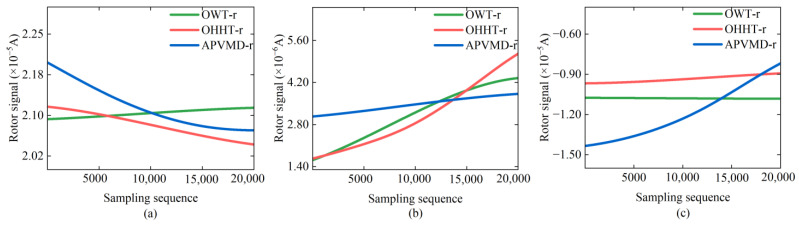
Trend term extraction results of the three sets of reconstructed signals: (**a**) trend term of Signal 1; (**b**) trend term of Signal 2; (**c**) trend term of Signal 3.

The high-frequency noise in the reconstructed signals has been significantly suppressed by implementing noise reduction processing on the rotor signals. In this case, the trend term of the reconstructed signals is closer to the force characteristics of the reverse torque in an ideal state. The extraction results of the trend term from the reconstructed signals processed by different noise reduction methods are displayed in Figure 8. As shown in Figure 8, the trend term of the reconstructed signals obtained by different methods generally follows the same pattern, indicating that the noise reduction algorithms primarily remove environmental noise without affecting the overall trend of the reverse torque. However, in Figure 8a, the trend term of OWT-r shows an abnormal change pattern, which may be due to the overlap of the frequency of external environmental noise with the rotor signal. During the OWT filtering process, not only is the noise removed, but the practical information of the reverse torque measurement may also be indiscriminately filtered, leading to a deviation in the trend term of the reconstructed signal.

**Figure 9:**
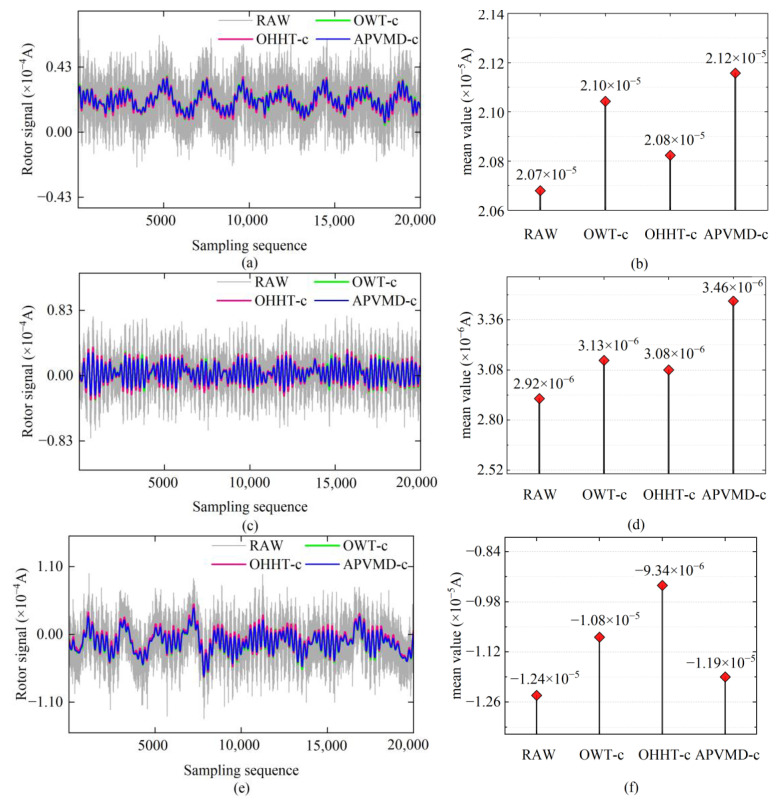
Results of error compensation for the reconstructed signal and the mean value change of the compensated signal: (**a**,**b**) Signal 1; (**c**,**d**) Signal 2; (**e**,**f**) Signal 3.

Using the mean value of the trend terms of the reconstructed signals in Figure 8 as the expected value of the ideal rotor signals, we performed error compensation on the three sets of reconstructed signals in Figure 7, obtaining the compensated signals shown in Figure 9 (marked as “method-c”). By comparing Figure 7 with Figure 9, it can be observed that since the compensation signals only corrected the overall offset of the reconstructed signals, they maintained consistency with the reconstructed signals in terms of local details. That is to say, APVMD-c still outperforms OWT-c and OHHT-c regarding smoothness. In the error compensation process, the amplitude of the upward and downward shifts of the reconstructed signals also varied due to the different mean values of the trend terms obtained by different algorithms. The parts b, d, and f in Figure 9 visually demonstrate the mean effect of the compensation signals obtained by different algorithms.

**Figure 10:**
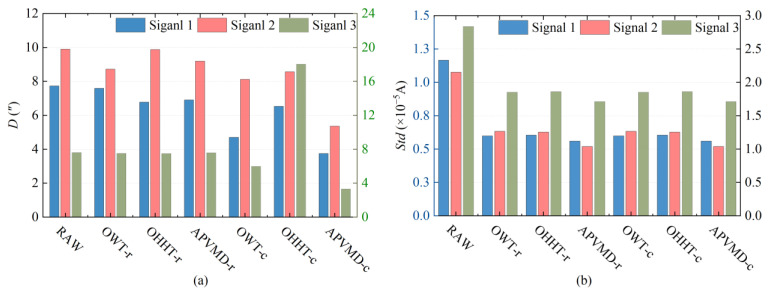
Evaluation of the noise reduction effect of the compensated signal: (**a**) changes in the *D* value metric; (**b**) changes in the *Std* metric.

Utilizing the OWT, OHHT, and APVMD algorithms, we respectively conducted noise reduction on three distinct sets of rotor signals and applied comprehensive offset error compensation to the reconstructed signals. Following this, we conducted a comparative analysis of the *D* and *Std* values for the signals that had been compensated using the various algorithms. Figure 10 illustrates the noise reduction outcomes for the rotor signals post-processing with each of the different algorithms.

From Figure 10a, the traditional gyro signal noise reduction models that adopt the “signal decomposition—selecting effective components for superposition” strategy, including OWT-r, OHHT-r, and APVMD-r, show relatively similar noise reduction performance. Compared to the original untreated rotor signal, the optimization range of the D-value for these three reconstructed signals is limited (within 2″). However, persistent external environmental interference often leads to significant *D* value deviations in azimuth measurement results, making the traditional gyro signal noise reduction model a key constraint factor that currently prevents significant improvements in azimuth measurement outcomes.

Compared to traditional noise reduction models, the OWT-c, OHHT-c, and APVMD-c algorithms can effectively reduce the *D* value, indicating that error compensation for the reconstructed signal is crucial for reducing the overall offset of the rotor signal. Among them, the APVMD algorithm significantly surpasses the other two methods in terms of improving noise reduction gain. However, the *D* value of OHHT did not decrease as expected and even showed varying degrees of increase, resulting in a negative gain for Signal 3. This occurrence is because the OHHT algorithm uses EMD in the signal decomposition process, which has issues with endpoint effects and mode aliasing, leading to the incomplete removal of noise from the reconstructed signal, and consequently making it difficult for the extracted trend term to reflect the expected value of the ideal rotor signal accurately. The shortcomings of the OHHT algorithm are particularly evident when processing the heavily noise-affected Signal 3, so the error compensation method for rotor signals based on OHHT needs further optimization. Similarly, OWT also faces similar issues to OHHT, including sensitivity to signal types and potential boundary effects, which may affect the accuracy of the trend term of the reconstructed signal.

Figure 10b shows that the noise reduction processing significantly reduced the dispersion of the rotor signals, effectively improving the smoothness of the signals, with a significant reduction in the *Std*. In particular, the compensated signal processed by the APVMD algorithm has a minor *Std*, proving the advantage of the algorithm proposed in this study in terms of stability and effectiveness.

### 4.3. Noise Reduction Results for All Sampled Rotor Signals

In Section 4.2, we conducted an exhaustive comparison between the traditional gyroscope signal denoising model (method-r) and the reconstruction signal offset error compensation model proposed in this study (method-c) in terms of their denoising effects on different types of rotor signals. The results show that by implementing offset error compensation on the reconstructed signals, we have partially overcome the difficulty of improving azimuth measurement error under continuous external environmental interference, thereby refocusing the emphasis of gyroscope signal processing on the research of signal decomposition algorithms. This shift has set more rigorous standards for the stability and effectiveness of denoising algorithms.

In view of this, to comprehensively evaluate the performance of the APVMD algorithm in the field of rotor signal denoising, we have not only employed traditional gyroscope rotor signal processing algorithms (OWT and OHHT) but also introduced GA and WOA that are also suitable for optimizing VMD parameters, to process the denoising of all 15 groups of rotor signals. Table 2 and Table 3 present the specific results of these denoising treatments in detail. The results show that, compared to the original rotor signals, after noise reduction and error compensation with the APVMD method, the *D* value decreased from an average of 7.06″ to 3.84″, reducing the azimuth measurement error by 45.63%; the *Std* of the rotor signals decreased from an average of 2.21 × 10^−5^ A to 1.19 × 10^−5^ A, reducing the dispersion of the compensated signals by 46.10%. This result further confirms that the APVMD algorithm is superior to the other four methods in terms of stability and effectiveness in rotor signal noise reduction.

According to Table 2, the OWT and OHHT algorithms achieved gains of 31.14% and 29.40%, respectively. However, OWT experienced three instances of negative optimization (i.e., the *D* value of the compensated signal exceeded that of the original rotor signal), and OHHT had five instances of negative optimization. The cause for this phenomenon is that the adaptability of the OWT and OHHT algorithms is inferior to that of the APVMD. Specifically, the performance of the OWT algorithm is largely limited by the characteristics of the signal itself. For signals with specific frequency distributions or transient features, OWT may not be the best option for processing rotor signals that are continuously subjected to external environmental interference. As for the OHHT algorithm, it has its own issues with endpoint effects and modal aliasing. Considering that rotor signals under continuous external interference contain a significant amount of noise, the OHHT algorithm is prone to generating false components when processing such high-noise signals, thereby affecting subsequent analysis work.

Additionally, the GVMD and WVMD algorithms achieved noise reduction gains of 33.84% and 37.56%, respectively, outperforming the OWT and OHHT algorithms. This advantage is mainly attributed to the robustness of the VMD algorithm, especially in processing non-stationary and nonlinear signals, where VMD can effectively extract the intrinsic modes of the signal from the noise. By combining GA and WOA to optimize the decomposition parameters of the VMD algorithm, the adaptability of the rotor signal decomposition process has been further enhanced. However, similar to the OWT and OHHT algorithms, the GVMD and WVMD algorithms also experienced negative optimization phenomena four and two times, respectively, indicating that GA and WOA are less accurate than the APSO algorithm in parameter optimization. This deficiency may be due to the higher complexity of the GA and WOA algorithm structures and their strong dependence on parameter settings, particularly when dealing with large sample data like rotor signals, making it difficult to fully leverage the potential of the algorithms.

According to Table 3, the dispersion of the rotor signals processed by different algorithms has significantly improved, with the *Std* decreasing by 40.69%, 40.41%, 44.74%, 45.78%, and 46.10%, respectively. Since the offset error compensation of the reconstructed signal does not alter its waveform characteristics, the improvement in internal accuracy is primarily related to the superposition and reconstruction of the effective signal components. Different noise reduction algorithms can effectively reduce the high-frequency noise in the sampled signals, resulting in the differences between the waveforms of the reconstructed signals obtained by each algorithm not being significant. Consequently, the standard deviations of the rotor signals after processing with different algorithms also show small differences. It is worth mentioning that the APVMD algorithm, due to its more precise signal decomposition capability, outperforms the other four algorithms in terms of the internal conformity accuracy of the noise-reduced signals.

## 5. Conclusions

This study proposes a noise reduction method that integrates the APVMD algorithm with error compensation for reconstructed signals, effectively reducing the measurement error of a maglev gyroscope under continuous external environmental interference.

(1) The parameters of the VMD model were optimized using the APSO algorithm, which outperformed the genetic algorithm (GA), whale optimization algorithm (WOA), and standard PSO in terms of convergence speed and optimization precision, making it suitable for parameter optimization of VMD for various types of rotor signals.
(2) The signal decomposition using the APVMD algorithm resulted in reconstructed signals with the lowest dispersion and accurate trend information. The corresponding compensation signals effectively corrected the overall offset error of the rotor signals. Field experimental data showed that the APVMD noise reduction method reduced the Std of the rotor signals by an average of 46.10% and improved the measurement accuracy of the north azimuth by an average of 45.63%. Its noise reduction performance surpassed that of the other four algorithms.

In future research, we will further evaluate the noise reduction performance of the proposed method under the interference of external vibration signals with different frequency characteristics. Furthermore, we intend to furnish preliminary data for signal decomposition parameters, tailored to the distinct features of rotor signals. This approach is expected to markedly decrease the computational time of the APVMD algorithm during azimuth measurement data processing, thereby broadening the utility of gyroscope sensors across a wider array of disciplines. Additionally, it is crucial to explore the impact of external environmental factors other than vibration interference (such as temperature changes) on the sampling signals of maglev gyroscopes. We will be dedicated to researching and establishing a compensation algorithm between the north-finding results and temperature differences to enhance the adaptability and reliability of maglev gyroscopes in complex application environments.

## Figures and Tables

**Table 1:** Multi-scale permutation entropy corresponding to IMF components.

	IMF1	IMF2	IMF3	IMF4	IMF5	IMF6	IMF7	IMF8	IMF9	IMF10
Signal 1	0.7545	0.7828	0.7822	0.7945	0.8056	0.8104	0.7835	0.8003	0.4169	--
Signal 2	0.7602	0.7833	0.7807	0.7769	0.7887	0.7889	0.7803	0.7936	0.4559	0.5048
Signal 3	0.7503	0.7804	0.7768	0.8108	0.7915	0.7969	0.7952	0.8105	0.5534	0.5525

**Table 2:** *D* values (″) corresponding to different noise reduction methods.

Scheme	RAW	OWT	OHHT	GVMD	WVMD	APVMD	Signal Type
Sets							
1	7.74	4.71	6.53	4.02	4.39	3.75	Periodic
2	9.91	8.12	8.57	--	6.18	5.36	Jitter
3	7.61	5.98	--	--	3.91	3.32	Mixed
4	6.53	3.87	4.26	4.38	3.64	3.18	Mixed
5	5.39	4.99	3.12	5.01	3.72	2.07	Jitter
6	6.97	2.62	5.41	2.48	3.09	2.12	Periodic
7	9.12	--	--	--	--	6.89	Jitter
8	6.47	--	4.07	5.41	4.58	3.04	Mixed
9	4.81	4.04	3.64	2.72	2.79	2.74	Mixed
10	8.34	4.94	5.07	5.83	5.22	4.87	Jitter
11	5.23	4.83	4.67	5.02	3.84	3.40	Mixed
12	7.89	6.07	3.57	2.94	4.03	2.26	Periodic
13	7.82	6.23	--	6.01	6.77	5.79	Periodic
14	8.29	3.19	--	--	--	6.41	Mixed
15	3.77	--	--	3.13	3.09	2.38	Periodic
**Number of effective denoise**	--	12	10	11	13	15	--
**Mean value**	7.06	4.97	4.89	4.27	4.25	3.84	--
**Improvement**	--	31.14%	29.40%	33.84%	37.56%	45.63%	--

**Table 3:** *Std* (×10^−5^ A) corresponding to different noise reduction methods.

Scheme	RAW Signal	OWT	OHHT	GVMD	WVMD	APVMD	Signal Type
Sets							
1	1.17	0.60	0.60	0.56	0.57	0.56	Periodic
2	2.15	1.27	1.26	1.04	1.05	1.04	Jitter
3	2.84	1.85	1.86	1.82	1.72	1.71	Mixed
4	1.77	1.37	1.37	1.35	1.35	1.34	Mixed
5	2.83	1.58	1.59	1.37	1.35	1.33	Jitter
6	2.30	1.26	1.26	1.24	1.24	1.23	Periodic
7	3.03	2.08	2.08	1.97	1.93	1.92	Jitter
8	2.39	1.69	1.69	1.65	1.55	1.54	Mixed
9	3.39	2.26	2.23	1.98	1.97	1.97	Mixed
10	1.85	1.05	1.06	0.81	0.80	0.80	Jitter
11	1.26	0.61	0.64	0.51	0.51	0.50	Mixed
12	1.94	1.06	1.07	1.06	1.07	1.05	Periodic
13	1.70	0.48	0.51	0.49	0.47	0.46	Periodic
14	2.73	1.55	1.57	1.53	1.45	1.44	Mixed
15	1.81	0.95	0.96	0.94	0.95	0.95	Periodic
**Mean value**	2.21	1.31	1.32	1.22	1.20	1.19	--
**Improvement**	--	40.69%	40.41%	44.74%	45.78%	46.10%	--

## Data Availability

The data that support the findings of this study are available upon reasonable request from the authors.
